# Contributions of Interactions Between Lifestyle and Genetics on Coronary Artery Disease Risk

**DOI:** 10.1007/s11886-019-1177-x

**Published:** 2019-07-27

**Authors:** M. Abdullah Said, Yordi J. van de Vegte, Muhammad Mobeen Zafar, M. Yldau van der Ende, Ghazala Kaukab Raja, N. Verweij, Pim van der Harst

**Affiliations:** 1 Department of Cardiology, University of Groningen, University Medical Center Groningen, 9700 RB Groningen, The Netherlands; 2 PMAS University of Arid Agriculture Rawalpindi, University Institute of Biochemistry and Biotechnology, 46000 Murree Road, Rawalpindi, Pakistan; 3 Genomics plc, Oxford, OX1 1JD UK; 4 Department of Genetics, University of Groningen, University Medical Center Groningen, 9700 RB Groningen, The Netherlands

**Keywords:** Genetics, Lifestyle, Coronary artery disease, Genetic lifestyle interactions, Genome-wide association studies, Genetic risk scores

## Abstract

**Purpose of the Review:**

To summarize current knowledge on interactions between genetic variants and lifestyle factors (G×L) associated with the development of coronary artery disease (CAD) and prioritize future research.

**Recent Findings:**

Genetic risk and combined lifestyle factors and behaviors have a log-additive effect on the risk of developing CAD.

**Summary:**

First, we describe genetic and lifestyle factors associated with CAD and then focus on G×L interactions. The majority of G×L interaction studies are small-scale candidate gene studies that lack replication and therefore provide spurious results. Only a few studies, of which most use genetic risk scores or genome-wide approaches to test interactions, are robust in number and analysis strategy. These studies provide evidence for the existence of G×L interactions in the development of CAD. Further G×L interactions studies are important as they contribute to our understanding of disease pathophysiology and possibly provide insights for improving interventions or personalized recommendations.

## Introduction

Coronary artery disease (CAD) is a complex multifactorial disease leading to ischemic heart disease and myocardial infarction. Globally, CAD is an important cause of death and morbidity, with approximately 9 million deaths between 2007 and 2017 [1]. In line with the most complex and non-communicable diseases, the development of CAD is the result of an interplay between both lifestyle and genetic factors [2••].

Lifestyle factors can broadly be defined as behaviors, customs, and habits of persons or groups, generally considered in the context of consequences for health [3]. Lifestyle risk factors are often modifiable and so are their risks. Interventions to adhere to a healthy lifestyle as a means of prevention have the potential to greatly reduce incident CAD event rates [4, 5].

Over the past decade, large advances in technology have moved the boundary of our understanding of the genetics of CAD. Genome-wide association studies (GWAS’s) and next-generation sequencing have helped to identify a large number of genetic loci associated with CAD and helped to better appreciate the complexity of its genetic architecture [6, 7••]. Despite our progress in the understanding of the complex genetics and the many lifestyle factors involved in the development and progression of CAD, the interplay between genetic variants and lifestyle factors leading to CAD remains largely obscure. Large-scale, well-powered studies investigating combined genetic and lifestyle risks have only recently started to fill this knowledge gap with credible evidence [2••, 8••].

In this review, we first summarize important knowledge of genetic and lifestyle factors associated with CAD and then focus on the contribution of genetic and lifestyle (G×L) interactions in the development of CAD. We conclude with future research directions to progress the field of G×L in CAD.

## Genetics of CAD

A heritable component to CAD has been well established, and recent studies estimate the heritability of CAD to range between 40 and 50 % [9]. Genetic analyses have been instrumental to progress our understanding of biological mechanisms involved in the development of CAD. Before the first well-powered GWAS in 2007, candidate gene studies were used to investigate common single nucleotide polymorphisms (SNPs) in genes coding for proteins with suspected biological importance in the pathophysiology of CAD. Although SNPs were frequently reported to be significantly associated with CAD, many candidate gene studies failed to achieve statistical significance after adjustment for multiple testing [10]. In addition, replication studies were often lacking. Since 2007, GWAS’s have become state-of-the-art to further our understanding of the genetics of complex diseases [6, 11]. GWAS’s investigate the association between millions of SNPs and a disease by comparing individuals with and without the disease [12]. In GWAS’s, common allele variants of SNPs that occur in at least 1–5% in the population are studied to determine their contribution to the disease [12]. To date, multiple GWAS’s have been performed on CAD in increasingly larger populations. Several SNPs have been identified to be strongly associated with CAD by both candidate gene and GWAS approaches, including *APOB* [13], *PCSK9* [7••], and *LPA* *[*7*••]*, which resulted in the development of drugs targeting these genes [14].

The most recent GWAS on CAD by van der Harst et al. included 122,733 cases and 424,528 controls and reported over 160 genome-wide significant (*P* < 5 × 10^−8^) loci associated with CAD. To understand the nature of these associations, possible shared genetic pathways with other traits or diseases were investigated and showed various associations with anthropometric measurements, lipids, inflammation markers, kidney function, diabetes mellitus, and blood pressure possibly providing an intermediate trait in the development of CAD [7••]. Insights into biology can also be obtained by studying gene expression patterns, for example with tools such as Data-driven Expression-Prioritized Integration for Complex Traits (DEPICT). DEPICT analyses on GWAS’s of CAD indicated important roles for platelets, blood vessel development, hemostasis, and a protein-protein interaction subnetwork [7••]. These findings provide reinforcement or novel evidence for the key roles of pathways and genes in the development of CAD and provide possible leads on how lifestyle factors might interact with them.

To further understand cumulative effects of biological pathways and outcomes associated with CAD, SNPs identified through GWAS’s have been summed to calculate genetic risk scores (GRS’s) as an estimation of an individual’s genomic risk of CAD. A weighted GRS counts the number of risk-increasing alleles (0, 1, or 2) per SNP for each individual and takes into account the effect size of each risk-increasing allele of each SNP as calculated in the GWAS. Since GRS’s are based on germline SNPs with alleles that are randomly allocated at conception, GRS’s are quantifiable from birth and potentially allow earlier risk stratification and primary prevention of events. GRS’s usually only include loci that reached genome-wide significance. For example, van der Harst et al. constructed a weighted GRS for CAD to investigate the risk of downstream cardiovascular diseases and observed associations with the development of atrial fibrillation and heart failure [7••].

Another strategy to estimate an individual’s genomic risk includes the creation of more extensive GRS’s using thousands or even millions of SNPs weakly or uncertainly associated with CAD. Another study constructed a CAD GRS with over 6 million SNPs and found individuals in the top 1% of the distribution were at almost 5-fold (odds ratio 4.83; 95% confidence interval, 4.25–5.46; *P* = 1 × 10^−132^) higher odds of CAD [15]. Inouye et al. used a GRS with 1.7 million SNPs linked to CAD and observed a 4-fold (hazard ratio 4.17; 95% confidence interval, 3.97–4.38) higher risk of CAD in individuals in the top quintile of the GRS compared with the lowest quintile [16]. A combination of the GRS with six conventional risk factors including diabetes, BMI, current smoking, hypertension, family history of heart disease, and high cholesterol led to a slight increase of 2.6% compared with the model with only the six conventional risk factors [16]. However, lipids and other biochemical variables were not available and a comparison with traditional risk scores such as the Framingham Risk Score could therefore not be performed [16].

So far, individual genetic variants identified by GWAS’s only explain ~ 15% of the estimated 40–50% heritability for CAD. The gap between currently explained CAD SNP–based heritability and other heritability estimates, the so-called missing heritability, may partly be found in rare variants which can be assessed using whole exome sequencing [17]. Using whole exome sequencing data, one recent study was able to increase SNP-based heritability estimates for height and BMI to pedigree heritability levels [18]. Rare variants in low linkage disequilibrium with neighboring variants, especially protein-coding variants, therefore likely contribute highly to heritability [18]. Another possibility is that underlying G×L interactions, in which the genetic component explains more variance depending on the lifestyle, remain to be elucidated. These G×L interactions could also possibly explain a proportion of the missing heritability.

## Genetics of Lifestyle Factors Associated with CAD

The importance of lifestyle factors in the development and primary prevention of CAD is well established. Rappaport et al. studied 3229 Swedish twins and estimated 21.6% of CAD deaths were attributable to non-modifiable genetic factors, and the remaining 78.4% to lifestyle and environment exposures during an individuals’ lifetime [19]. The INTERHEART study investigated the importance of modifiable risk factors in 52 countries across the globe and found that raised apolipoprotein B/A1 ratio, current smoking status, no regular alcohol intake, hypertension, diabetes, abdominal obesity, psychosocial factors (depression, low locus of control, perceived stress, and major life events), lack of daily fruit and vegetable consumption, and no regular physical exercise accounted for most of the risk of acute myocardial infarction [20]. Although the identified lifestyle factors independently increase CAD risk, lifestyle risk factors tend to cluster in adults, with 20% of the individuals of the general population having at least three lifestyle risk factors [21]. The lifestyle factors highlighted by the INTERHEART study are discussed below.

### Tobacco Smoking

Tobacco smoking is a major risk factor for CAD [22]. Since the average cigarette contains a complex and changing mix of poisonous compounds with various pathological effects [23], the exact mechanisms leading to CAD remain unknown [24]. Currently, it is known that smoking leads to atherosclerosis through endothelial dysfunction and damage, plaque vulnerability with increased risk of rupture, increased inflammatory and thrombotic state, and increased blood pressure [24]. Tabaco smoking is also under the influence of genetic factors, including several SNPs associated with smoking initiation, heaviness, and cessation [25, 26].

### Alcohol and Coffee Consumption

Heavy alcohol consumption has been described to increase risk of CAD, whereas low to moderate intakes might reduce the risk [27, 28]. Similar to smoking, genetics also influence alcohol intake [26]. Liu et al. found 99 SNPs associated with the amount of drinks per week, but did not find a significant genetic correlation between drinks per week and phenotypic CAD [26].

A similar U-shaped risk pattern has been described for coffee consumption: excessive or no observational coffee and caffeine intake are associated with increased risks for CAD risk while moderate intakes appear to reduce risk [29, 30]. Genetic studies did not yield evidence for causal links between caffeine intake and CAD [29, 31], suggesting that reported beneficial observational findings may be confounded by the numerous non-caffeine constituents of coffee [30].

### Physical Activity and Sedentary Behavior

Physical activity plays an important role in both the primary and secondary prevention of CAD [32]. Epidemiological studies show a dose-response relationship leading to a 20% reduction of cardiovascular events in individuals who practice leisure-time physical activity. Moreover, in secondary prevention, exercise training has been shown to improve endothelial function, halt the progression of coronary stenosis, and possibly induce collateral formation leading to improved myocardial perfusion [32]. However, despite these known protective effects, there is a dangerous trend towards less physical activity worldwide [33]. In addition, sedentary behavior has been established as an important driver of chronic diseases, independent of physical activity levels [34]. Although twin and family studies showed that both physical activity and sedentary behaviors are potentially heritable [35], GWAS’s were mostly performed on self-reported data of physical activity, yielding only a few associated loci [36]. A recent GWAS using accelerometer data found one locus associated with overall activity and 4 loci with sedentary behavior, explaining up to 21% of the heritability of physical activity and 12.9% of sedentary behavior [37]. Given the large economic and health burden [38, 39], more research into the genetic architecture of physical activity and sedentary behavior is needed.

### Diet

The impact of diet on CAD has been studied mostly in observational studies, which have generally found Mediterranean and DASH (Dietary Approaches to Stop Hypertension) diets, which are both rich in fruits, vegetables, and nuts, to be associated with lower risk of MI and improved cardiometabolic factors with effects on blood pressure, blood lipids, inflammation, endothelial function, and thrombosis [40]. Robust genetic data to further understand the determinants of interindividual variation in response to diet is largely missing, although diet may influence epigenetic changes such as DNA methylation [40, 41].

Lifestyle behavior, especially smoking and alcohol use, can also be viewed as the willingness of an individual to take certain health risks. This risk tolerance or behavior may have a predisposing genetic architecture, as shown by Karlsson Linnér et al. who found hundreds of loci associated with risk tolerance and risky behaviors, including 124 SNPs associated with general risk tolerance [42]. The general risk tolerance SNPs were associated with genes that were highly expressed in brain regions such as the prefrontal cortex, basal ganglia, and midbrain [42].

## Gene × Lifestyle Interactions

Genetic and lifestyle factors both contribute to each person’s risk of CAD and have a complex interplay. Lifestyle factors themselves are partly determined by genetic factors. In addition, increasingly more evidence indicates lifestyle can also modify the effects of genetic variants on CAD. This may be due to shared etiological pathways between the genetic variant and lifestyle risk factor, in turn leading to the disease. Another potential mechanism includes epigenetics, in which gene expression is altered by lifestyle or environmental factors through local chromatin environment changes affecting DNA accessibility despite the absence of DNA alterations [41, 43].

Some of the complex gene and lifestyle interplay can be better understood through G×L interaction studies. These interactions can be investigated using several approaches, as discussed below.

### GRS Approach

**Fig. 1:**
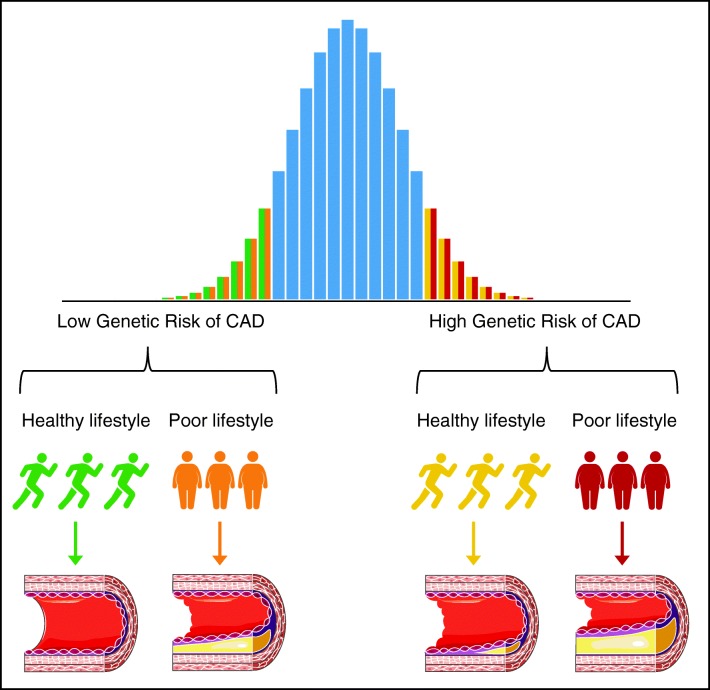
Combined genetic and lifestyle risks increase the risk of coronary artery disease. The risk of coronary artery disease (CAD) in individuals with low or high genetic risk is higher amongst individuals with poor lifestyle compared to a healthy lifestyle. Compared with individuals with low genetic risk, individuals with high genetic risk start off at higher risks of CAD, with the highest risks of CAD amongst individuals with poor lifestyle and high genetic risk

It is possible to construct a GRS based on the most highly associated variants of previous GWAS’s and analyze the interaction between this overall genetic risk and a lifestyle factor on CAD. However, the number of studies investigating the combined risks of both genetic and lifestyle factors is little. To date, two large studies investigated the combined risks of CAD associated with genetics and overall lifestyle [2••, 8]. The first study by Khera et al. included 55,685 individuals with 5,103 (9.2%) cases from four cohorts [8••], and the second study by Said et al. included 325,113 individuals with 9,771 (3.0%) cases from the UK Biobank [2••]. Both studies calculated a weighted GRS based on previous reports and categorized the population into quintiles of genetic risk. The lowest quintile was taken as a low genetic risk, the second to fourth quintile as intermediate risk, and the highest quintile as high genetic risk. Lifestyle was subsequently categorized as ideal, intermediate, or poor [44]. Individuals with poor lifestyle were at higher risk of CAD compared with individuals in the same genetic risk category but with an ideal lifestyle. The risk of CAD increased not only with less than ideal lifestyle, but also with increasing genetic risk. Importantly, both studies showed individuals with ideal lifestyle and high genetic risk were at nearly twice the risk of developing CAD compared with individuals with an ideal lifestyle but low genetic risk. These findings indicate individuals with high genetic risk have a higher starting risk of developing disease compared with individuals with similar lifestyle but lower genetic risk, and even higher risks of events if they have a poor lifestyle and high genetic risk (Fig. 1).

Although neither study observed statistically significant interactions between lifestyle categories and genetic risk, Said et al. estimated that with 80% power and an alpha of 0.005, interaction effects between genetic risk and intermediate or poor lifestyle would range from 1.21 to 1.50 [2••]. The risk of CAD in individuals with high genetic risk therefore possibly not only starts off at higher risk, but also increases more strongly with worse overall lifestyle (Fig. 1).

The importance of physical activity in genetic risk groups of CAD has been reported as well [45]. One study found that higher grip strength and cardiorespiratory fitness were associated with a lower risk of incident CAD events across tertiles of genetic risk of CAD [45].

### Genome-Wide Interaction Studies

Instead of selecting lead variants of previous GWAS’s and testing these for interactions with lifestyle factors, SNPs across the whole genome can be scanned in a genome-wide interaction study. Because unveiling G×L interactions requires well-powered studies, the CHARGE Gene–Lifestyle Interactions Working Group was formed [46]. To date, this has led to three studies on the interaction between lipid levels with smoking [47•], physical activity [48•], and alcohol intake [49•]. These studies used a joint meta-analysis in which 2-degree-of-freedom test was adopted that jointly evaluates interaction and main effects to increase statistical power [50]. Bentley et al. studied the interaction between lipid levels with smoking and revealed 13 new loci associated with lipids, of which several appeared to be driven mainly by their interaction with smoking [47•]. The importance of interaction testing was further highlighted by another study in which 4 novel loci were discovered when testing for interactions between genetically determined lipid levels and physical activity, whereas not a single new locus was found in the test without interaction [48•]. The third study revealed 18 novel lipid loci, although none of which appeared to be driven by interactions with alcohol intake [49•]. Based on these studies, smoking and physical activity, but possibly not alcohol use, may be modifiable lifestyle risk factors of interest to alter blood lipid levels. Even if these interactions contribute little to the overall variance of blood lipid levels, insights in these interactions could contribute to our understanding of disease pathophysiology.

### Twin Studies

One twin study assessed G×L interactions amongst 51,065 Swedish same-sex twins. This study found higher BMI was associated with lower genetic variance of CAD, suggesting a more important role for genetics in the development of CAD in individuals with low BMI [51]. Other lifestyle factors such as smoking and sedentary behaviors showed no significant G×L interactions, but rather seemed to increase CAD risk directly [51].

### Candidate Gene Studies

The largest candidate gene study on G×L interactions is a meta-analysis which investigated the interaction between 45 CAD loci and smoking in 60,919 cases and 80,243 controls. In this study, a significant interaction was observed between rs7178051, located within *ADAMTS7*, and smoking [52]. Several studies including up to ~ 6,000 individuals reported interactions between *ApoE* and smoking as well [53–55]. However, larger meta-analyses did not find support for this conclusion [52, 56]. This stresses the need for large sample sizes in and replication of candidate gene studies investigating G×L interactions [57]. Many other candidate gene studies tested the interaction between CAD genes and lifestyle risk factors, including smoking [58–65], alcohol intake [62, 65, 66], diet [67–69], and physical activity [69]. However, these results should be interpreted with caution as sample sizes were small and results were not replicated in independent larger studies.

## Future Perspectives

Only recently, studies with sufficient sample sizes have emerged and reported robust G×L interactions. Although the effect sizes are small and might add little to the explained variance of CAD heritability, they increase our knowledge on complex G×L interplays [15, 16]. This knowledge might be translated to strategies that pinpoint lifestyle risk factors with proven interactions and are therefore of increased interest to modify. Next, in the current era of huge biobanks, several cohorts will be well powered to perform genome-wide interaction analyses on CAD and modifiable risk factors of interest in the years to come.

In line with most GWAS’s, the GWAS’s on CAD included only or mostly white Europeans in order to reduce heterogeneity [70]. Logistic, systemic, or historical factors that make it easier to perform genetic studies in Europeans play a role as well [70]. As a consequence, little attention has been paid to genetic variants in other populations. Variants associated with G×L interactions may have different effects in non-Europeans, reducing its broader applicability in the clinic. As long as there is a lack of well-powered cohorts in other ethnicities, candidate gene studies offer an excellent approach to validate genes known to interact with lifestyle risk factors [71].

## Conclusion

In this review, we summarized current knowledge on the genetics of CAD, lifestyle factors, and the genetics of lifestyle factors associated with CAD. We focused on the interplay of genetics and lifestyle, especially the effect modifications as determined by G×L interaction studies. The majority of G×L interaction studies are small-scale candidate gene studies, lacking replication and therefore providing spurious results. Only few studies are robust in number and analysis strategy. These studies provide evidence of the existence of G×L interactions. Current data suggest that genetics and G×L interactions contribute little to the overall risk prediction for CAD next to lifestyle and other phenotypic risk factors. However, well-powered G×L interactions studies are important as they contribute to our understanding of disease pathophysiology and may provide insights into improving interventions or personalized recommendations.
